# A Clinical Study of Intraoperative Perfusion Chemotherapy in Gastric Cancer: A Prospective Cohort Study

**DOI:** 10.7759/cureus.58482

**Published:** 2024-04-17

**Authors:** Sohail Ahmed, Muhammad Amir, Khan Adnan, Zhang Zilong, Amna Akbar, Sania Khan, Sarosh Khan Jadoon, Mohammad Saleem Khan

**Affiliations:** 1 Gastrointestinal Surgery, Yangtze University, Jingzhou, CHN; 2 Emergency, Midland Doctors Medical Institute, Muzaffarabad, PAK; 3 Oncology, Jingzhou Central Hospital, Jingzhou, CHN; 4 Surgery, District Headquarter Hospital, Jhelum Valley, Muzaffarabad, PAK; 5 Oncology, Shaukat Khanum Memorial Cancer Hospital and Research Centre, Lahore, PAK; 6 General Surgery, Combined Military Hospital, Muzaffarabad, PAK; 7 Medicine, District Headquarters Hospital (DHQ) Teaching Hospital, Kotli, PAK

**Keywords:** chemotherapy, drug efficacy, gastric cancer, intraoperative chemotherapy, lobaplatin

## Abstract

Introduction: Gastric cancer (GC) is the third largest cause of cancer-related death worldwide, with major geographic disparities in incidence and outcomes. Sociodemographic indicators, food habits, and genetic predispositions all add to the load. Despite advances in systemic treatments, peritoneal metastasis remains a concern, with intraperitoneal chemotherapy (IPC) emerging as a promising treatment option.

Methods: A prospective cohort research was done, with 30 GC patients receiving cytoreductive surgery (CRS) followed by lobaplatin-based intraoperative chemotherapy. The study evaluated postoperative complications, survival rates, and disease recurrence using Statistical Package for the Social Sciences (SPSS) version 25.0 (IBM SPSS Statistics, Armonk, NY) for data analysis. The purpose of this study is to assess the effectiveness, safety, and dependability of lobaplatin as an intraoperative chemotherapeutic agent in patients having gastric cancer surgery, with a particular emphasis on those patients who do not have distant metastases.

Results: The study population had a balanced gender distribution, with an average age of 44.83 years. Most patients had advanced-stage cancer (T3 and T4), and lobaplatin treatment resulted in a low frequency of serious postoperative sequelae. Preliminary studies suggest that lobaplatin is a safe and potentially effective IPC drug for GC, with few side effects and adequate survival rates.

Conclusion: Lobaplatin shows promise as an intraoperative chemotherapeutic treatment for gastric cancer, necessitating more research in bigger, randomized controlled studies to determine its efficacy and safety profile. The study emphasizes the need for novel treatment strategies to enhance the prognosis of GC patients, particularly those with peritoneal involvement.

## Introduction

According to World Cancer Research Fund International (WCRF), stomach cancer is the third most frequent cause of mortality related to cancers. Up to 40% of stage III gastric cancer (GC) has peritoneal metastasis [1]. According to the ninth edition of the American Joint Committee on Cancer (AJCC), gastric carcinomas are staged as cancers found within the gastric cardia, and tumors that affect the esophagogastric junction (EGJ) and have a central point located more than 2 cm into the upper part of the stomach are now classified as gastric carcinomas [2]. Formulating effective preventative strategies requires an understanding of the current stomach cancer burden and the differences in trends across different locales. Over 1.22 million incident cases of stomach cancer were reported globally in 2017, accounting for 19.1 million disability-adjusted life years (DALYs). Since 1990, there has been a drop in the global age-standardized rates of stomach cancer (incidence, deaths, and DALYs), despite an increase in absolute numbers. A better sociodemographic index was linked to the decline in the burden of disease. Worldwide, smoking in males was responsible for 24.5% of the age-standardized DALYs, whereas a high-sodium diet contributed 38.2% of the DALYs in both sexes combined [3].

East Asian countries, which include Korea (32.5), Japan (32.5), and Mongolia (32.5), have the greatest rate of incidence of the disease, with one case per 100,000 people in 2020. On the other hand, Africa and North America have been reported to have low incidence rates. Due to its substantial population, China has accounted for approximately 44% of the global cases of stomach cancer. The male/female ratio was 2.4, and there was heterogeneity in survival rates worldwide. The highest survival rates were reported in South Korea (68.9%) and Japan (60.3%). One-year survival rates in Austria, Germany, the United Kingdom, and China ranged from 13% to 41%. In the United States, the rate of occurrence of GC is greater among Latinos compared to non-Hispanic White people. In China, the rate of occurrence was significantly greater in rural regions compared to urban regions (22.82 versus 17.29). Those with older age, male gender, and diffused tumors have poor survival [4].

Stomach cancer risk is elevated by the presence of inherited mutations in specific genes, such as the *CDH1* gene or GSTM1-null phenotype. Gastric adenocarcinoma and proximal polyposis of the stomach (GAPPS) is a hereditary disorder characterized by an increased risk of development of stomach cancer. Bacterial infection with *Helicobacter pylori* is the primary causative agent in the link between gastric ulcer and stomach cancer [5]. Researchers have also shown that long-standing gastroesophageal reflux disease (GERD), tobacco consumption, excessive alcohol intake, exposure to dust at work, high-temperature particles, and metals such as chromium VI [6], talc [7], and asbestos all have been linked with increased incidence of gastric cancer. Populations with diets abundant in sodium and preserved foods containing high levels of N-nitroso compounds, such as the Japanese, demonstrate elevated incidences of stomach cancer. Less common risk factors include obesity [8], pernicious anemia, previous gastric surgery, ionizing radiation, Epstein-Barr virus, low socioeconomic status, and blood group type A [5].

Inadequate fresh food storage, poor water quality in developing countries, and being Black males are risk factors. The lowest rates were reported in White individuals, the second and third generations born in the United States, and higher income groups. Japanese migrants have shown evidence of nutritional, social, and medical influences, rather than a genetic predisposition. The histological patterns of stomach cancer have undergone a shift; stomach cancer is shifting from the distal to the proximal region. The intestinal gastric type is more prevalent (70%) and is affected by age more than 50 years and environment. In contrast, the diffuse or infiltrative type is less common (30%) but is identified at a younger age in both males and females and has a poorer prognosis [9].

The inherently aggressive characteristics of stomach cancer, along with historically unfavorable results even in cases when the disease is operable, have resulted in the gradual development of the idea of survivorship. Due to the dearth of trials available for peritoneal carcinoma (PC) patients, chemotherapy has remained the cornerstone of treatment even in the absence of sufficient data on its effectiveness. It is postulated that intravenous chemotherapy's capacity to infiltrate and eradicate peritoneal cancer cells is restricted by insufficient transfer across the blood-peritoneal barrier. Modifications to peritoneal function gradually impair its ability to perform the efficient exchange of fluids, causing ascites, obstruction of the bowel, and discomfort that ultimately results in death [10].

Studies have demonstrated that systemic treatment leads to enhanced survival and increased quality of life when compared to receiving the best supportive care. For patients with advanced stomach cancer who are in good health, it is advised to consider using combinations of platinum and fluoropyrimidine, such as doublet or triplet regimens. Nevertheless, it is crucial to always evaluate comorbidities, organ function, and performance status (PS) [11]. Intraperitoneal chemotherapy (IPC) is a procedure in which a chemotherapeutic agent is directly poured into a patient's peritoneal cavity. In this way, the drug attains markedly elevated local concentrations, approximately 1,000-fold greater than the highest levels found in the bloodstream. Hyperthermia enhances the effectiveness of chemotherapy by enhancing the penetration of drugs into tumor masses and their uptake by cancer cells. In order to get the best possible survival outcome, IPC should be used in conjunction with established systemic chemotherapy [12].

Systemic and IP chemotherapy (SIPC) is a type of bidirectional chemotherapy wherein an implantable catheter system is used to combine systemic chemotherapy with IPC. Although SIPC morbidity was low, there were conflicting findings regarding its effectiveness. Delivered through an IP port inserted during surgery, IPC has also been studied as an adjuvant therapy after radical surgery, with reported benefits in a specific patient population. Pressurized intraperitoneal aerosol chemotherapy (PIPAC), an additional IPC technique with the potential for better peritoneal delivery, has been used primarily in palliative indications and is the focus of active research now [13]. Studies have demonstrated the efficacy of intraoperative intraperitoneal chemotherapy (IPC) and extended intraoperative peritoneal lavage (EIPL) as viable treatment options [14]. The most recent consensus guidelines (National Comprehensive Cancer Network (NCCN)/European Society for Medical Oncology (ESMO)/Japanese) do not currently advise intraperitoneal chemotherapy outside of clinical trials [15]. As per the Korean Practice Guidelines for Gastric Cancer 2022, the use of intraperitoneal (IP) chemotherapy in gastric cancer is recommended solely for research purposes [16].

## Materials and methods

The study aimed to assess the reliability, efficacy, and safety of lobaplatin in intraoperative chemotherapy of gastric cancer surgery. Patients of age more than 18 years and less than 90 years with no other terminal illness who had undergone the basic diagnosis process were included in the study. Availability of clinical data such as blood tests, endoscopy, biopsy, and laparoscopy and absence of distant metastasis were the inclusion criteria. The approval for the study was given by the Ethical Committee of Jingzhou Central Hospital, China, under IRB number 2023L20003/YZEDU/2023, and data was collected from the same.

Polytrauma patients with concurrent confounding pathologies (tumor, renal failure, liver cirrhosis, tetraplegia, infection (diskitis, osteomyelitis or epidural abscess, and tuberculosis), and artificial disc) were excluded. Pregnancy or other medical conditions, patients previously operated for gastric or other conditions, and the presence of distant metastasis to the brain were also exclusion criteria. Keeping in view the abovementioned criteria, a total of 30 patients were included in this study.

After conducting a comprehensive history-taking and clinical examination of the patients and obtaining their informed consent, we determined whether they would take part in the study. Each clinicopathological characteristic was documented carefully. All patients underwent comprehensive observational and clinical evaluation before undergoing any kind of treatment. Patient characteristics and tumor features (such as tumor size, lymphatic system, metastases, histological evaluation, and subtypes) were investigated. The American Joint Committee on Cancer (AJCC) categorization system was used to identify the clinical stages of cancer in people who were eligible for inclusion. After performing cytoreductive surgery (CRS) for gastric cancer, lobaplatin was used as the chemotherapeutic drug for intraoperative chemotherapy. It was given with a dose of 50 mg/m^2^ dissolved in 3,000 mL 5% IV glucose solution. The solution was heated and maintained at 43 degrees centigrade. It was pumped into the peritoneal cavity at a rate of 500 mL/minute via the left and right upper abdominal drainage tubes. It was then allowed to escape the abdomen via the left and right lower abdominal drainage tubes. The total time for the procedure was 60 minutes. Each patient was noted for postoperative complications. These included high-grade fever, allergic reaction, nausea/vomiting, intestinal obstruction, abdominal bleeding, abdominal infection, surgical site infection, abnormal liver function tests (LFTs), abnormal renal function tests (RFTs), lung infection, neurotoxicity, and peritoneal recurrence. No patient died during or after the procedure.

The preformat form was created to incorporate all relevant factors specified earlier in the clinicopathological features. The Statistical Package for the Social Sciences (SPSS) version 25.0 (IBM SPSS Statistics, Armonk, NY) was utilized to carry out data analysis. Both qualitative and quantitative variables are provided as descriptive statistics. In this study, frequency and percentages were used to measure factors such as gender, American Society of Anesthesiologists (ASA) score, T-stage, lymph node status, tumor clinical stage, and postoperative complications (high-grade fever, allergic reaction, nausea/vomiting, intestinal obstruction, abdominal bleeding, abdominal infection, surgical site infection, abnormal LFTs, abnormal RFTs, lung infection, neurotoxicity, and peritoneal recurrence). The mean and standard deviation (SD) values for quantitative factors such as age and body mass index (BMI) were calculated. It is generally accepted that if p is less than 0.05, a substantial correlation exists. An examination of the correlation between lobaplatin as an intraperitoneal chemotherapy drug and various clinical and pathological features was conducted in the context of prospective cohort research over a period of two years (December 2021 to April 2023). A total of 150 cases of gastric cancer were reported to the hospital in two years, but we used a sampling method that did not rely on probability and included 30 patients in the final analysis of the data. The standards outlined in the Strengthening the Reporting of Observational Studies in Epidemiology (STROBE) statement were adhered to in the presentation of research findings. This ensured that the data were interpreted correctly and completely.

## Results

Data was analyzed for all variables using SPSS version 25.0 software. Frequency and percentages were determined for variables such as gender, American Society of Anesthesiologists (ASA) score, T-stage, lymph node status, tumor clinical stage, and postoperative complications (high-grade fever, allergic reaction, nausea/vomiting, intestinal obstruction, abdominal bleeding, abdominal infection, surgical site infection, abnormal LFTs, abnormal RFTs, lung infection, neurotoxicity, and peritoneal recurrence). The mean and standard deviation values for quantitative factors such as age and BMI were calculated.

A summary of the clinical parameters of patients who underwent intraoperative chemotherapy is given in Table 1.

**Table 1:** Clinical parameters of patients who underwent intraoperative chemotherapy ASA: American Society of Anesthesiologists, SD: standard deviation, BMI: body mass index

Variables	Frequency (percentage)
Gender	
Male	18 (60)
Female	12 (40)
ASA score	
1	12 (40)
2	12 (40)
3	6 (20)
T-stage	
T3	17 (56.7)
T4	13 (43.3)
Lymph nodes	
N0	4 (13.3)
N1	5 (16.7)
N2	4 (13.3)
N3a	9 (30)
N3b	8 (26.7)
Clinical stage	
III	14 (46.7)
IV	16 (53.3)
Variable	Mean±SD
Age (range, years)	44.83±12.5 (19-90)
BMI (range, kg/m^2^)	33.3±7.3 (21-46.3)

The age range for our population was 18-90 years. The mean age of our study population was 44.83, and a median age of 40.50 years was obtained. The BMI range of our population was 21-46.3 kg/m^2^. The mean BMI for our study population was 33.31, and the median was 32.45. In our study, there were a total of 30 patients. Of the 30 patients, 18 were male, constituting 60% of the population, and 12 were female, constituting 40% of the population. In patients undergoing cytoreductive surgery (CRS) followed by intraperitoneal chemotherapy, 12 (40%) had an ASA score of 1, 12 (40%) had an ASA score of 2, and six (20%) had an ASA score of 3.

Our study included patients only with T3 and T4 tumor stages. In our population, 17 patients had T3 stage, constituting 56% of the population, and the remaining 13 patients had T4 stage, constituting 43% of the population (Table 1).

The most prevalent lymph node involvement in our study population was N3a (30%). Lymph node involvement was not observed in only four (13.3%) patients. Of 30 patients, 14 (46.7%) were at stage III, and of 30 patients, 16 (53.3%) were at stage IV.

Postoperative complications

**Figure 1:**
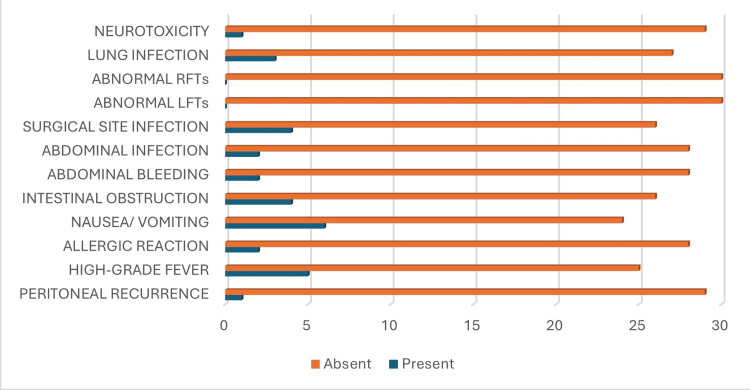
Frequency of postoperative complications RFTs: renal function tests, LFTs: liver function tests

Several postoperative complications were seen after cytoreductive surgery (CRS) followed by intraperitoneal chemotherapy. These are summarized in Figure 1.

Neurotoxicity after CRS + intraoperative chemotherapy was observed in only one (3%) out of 30 patients in our study. Lung infection was diagnosed in only three (10%) patients, while the remaining 27 (90%) patients were not infected. None of the patients in our study population developed acute kidney injury or acute liver injury. Four (13.3%) out of 30 patients in our study population developed surgical site infection. Abdominal infection developed in two (7%) out of 30 patients in our study population, whereas the remaining 28 (93%) patients remained infection-free. Abdominal bleeding was seen in two (7%) out of 30 patients in our study population. Four (13%) out of 30 patients in our study population developed intestinal obstruction, while the remaining 26 (87%) did not. Nausea/vomiting was the most common postoperative complication seen in our study. Out of 30 patients, six (20%) developed nausea/vomiting. Allergic reaction/hypersensitivity reaction developed in two (7%) out of 30 patients in our study population, whereas the remaining 28 (93%) patients remained normal. In our study population, high-grade fever was documented in only five (17%) patients, while the remaining 25 (83%) patients remained afebrile. After CRS + IPC, only one (3%) out of 30 patients in our study developed peritoneal recurrence (Figure 1).

## Discussion

The treatment of gastric cancer (GC) depends on the stage of the cancer and the patient's characteristics. Palliative gastrectomy is necessary for some patients with abdominal implantation metastases because of tumor-related obstruction, bleeding, and perforation. Intraperitoneal hyperthermic perfusion chemotherapy (IHPC) with early sequential is one alternative. Drugs used in intraperitoneal chemotherapy, however, can only reach a depth of less than 2 mm below the tumor's surface. Therefore, multimodal therapeutic techniques, such as IHPC combined with cytoreductive surgery (CRS), are suggested [1]. In Western countries, the mainstay of treatment for peritoneal metastasis is hyperthermic intraperitoneal chemotherapy (HIPEC) with platinum derivatives with or without mitomycin C (MMC). In Asian countries, on the other hand, either a mix of HIPEC and normothermic intraperitoneal (IP) chemotherapy is preferred, or they provide repeated doses of IP chemotherapy through IP port systems [17]. The choice of therapeutic drug is important for IPC. One study suggested the use of paclitaxel (PTX) or docetaxel (DTX), MMC as monotherapy, a combination of MMC and cisplatin, or a combination of cisplatin and fluorouracil in cases of positive peritoneal cytology or T3-T4 or peritoneal metastasis [18].

Cisplatin and MMC are commonly employed in IPC due to their ability to enhance the effects of heat and their resistance to thermal degradation. Taxanes, including paclitaxel (PTX) and docetaxel (DTX), are well-suited for IPC due to their hydrophobic nature and large molecular weight. Taxanes are particularly beneficial for patients with peritoneal carcinomatosis (PC) and a significant tumor burden. Furthermore, the recurrent administration of PTX and DTX only leads to modest fibrotic adhesions in the peritoneum [19]. A meta-analysis demonstrated that the addition of IPC to surgery resulted in an enhanced five-year survival rate (risk ratio: 3.10) and a decreased risk of recurrence (odds ratio: 0.45) compared to surgery alone. In Japan, CY1P0 patients are more inclined to have preoperative IPC in conjunction with radical D2 gastrectomy [15]. HIPEC may stop a peritoneal recurrence and may increase survival time. In 1,145 patients from 11 studies undergoing potentially curative gastrectomy but do not have clinically evident metastases or positive peritoneal cytology, morbidity (17%-60% versus 25%-43%), overall survival (OS) (32-35 months versus 22-28 months), five-year survival rates (39%-87% versus 17%-61%), and peritoneal recurrence (7%-27% versus 14%-45%) was significantly different in prophylactic HIPEC and surgery alone [20].

Another meta-analysis of 32 trials encompassing 2,520 patients compared HIPEC and conventional oncological management for the treatment of advanced-stage gastric cancer with and without peritoneal carcinomatosis (PC). The outcomes were emphasized and examined in detail when HIPEC was applied as a preventative or remedial measure. The HIPEC group had higher overall survival rates at three or five years in patients without PC. A four-month increase in median survival was seen in PC patients, with the HIPEC group benefiting from this improvement. On the other hand, a much higher incidence of respiratory and renal failure was linked to HIPEC. The findings show that HIPEC has a survival benefit in certain patient groups when used as a preventative and therapeutic measure [21]. The GASTRIPEC-I trial, a multicenter randomized controlled trial (RCT) examining the effect of HIPEC on survival following CRS, was published in 2021. When comparing the CRS-only arm (53 patients) to the CRS plus HIPEC arm (52 patients), the median survival was similar (14.9 months, p=0.16); however, the combination arm showed significantly better progression-free survival and metastasis-free survival [22].

In a meta-analysis of eight trials, one group used a combination of docetaxel and cisplatin; the other seven groups used a single IP drug (paclitaxel or docetaxel). Paclitaxel (dose of 20-80 mg/m^2^), cisplatin (dose of 30 mg/m^2^), and docetaxel (dose of 30-60 mg/m^2^) were the most prescribed dosages. Normothermic IP chemotherapy demonstrated encouraging outcomes in multiple phase II and non-randomized retrospective studies, with patients with gastric cancer and PM or positive cytology showing a median overall survival of 12 months and 30.5 months. This idea of neoadjuvant intraperitoneal and systemic chemotherapy (NIPS) is supported by the response rates, which ranged from 22% to 97% in the bidirectional neoadjuvant setting [23].

Another study used a combination of oxaliplatin and 5-fluorouracil for IPC. Three (6.7%) patients in the IHPC group and one (2.2%) patient in the control group had a substantial decrease in peripheral blood leukocytes. The two patient groups had incidence rates of abdominal pain of 35.6% and 28.9%, respectively. Statistically significant variations in postoperative complications were not found in both groups. Blood profile and liver and kidney function were also similar. Nevertheless, serum creatinine (S Cr) and blood urea nitrogen (BUN) levels were significantly different in both the IHPC group and the control group (p<0.05) [24].

In a meta-analysis involving 1,906 patients in 16 RCT studies, it was reported that the combination therapy (surgery plus IHPC) significantly improved survival and reduced recurrence rate compared to surgery alone. There was no significant increase in the risk of myelosuppression, ileus, anastomotic leakage, bowel perfusion, or hypohepatia. Survival rates were significantly increased at one to nine years, and recurrence rates were significantly lower at two, three, and five years in the combination therapy group [1]. Lobaplatin has antitumor activity, which is mainly due to the conformation of DNA-drug adducts, mainly as AG and GG intra-strand cross-links. The expression of the *c-Myc* gene, which is linked to oncogenesis, apoptosis, and cell proliferation, was previously shown to be impacted by lobaplatin [20]. Lobaplatin is known to inhibit the growth of human gastric cancer cells and trigger apoptosis. This process has been linked to increased expression of Bax, poly (ADP-ribose) polymerase (PARP) cleavage, p53 expression, and decreased expression of Bcl-2 [25]. Very limited data is available on the use of lobaplatin in intraperitoneal chemotherapy in gastric cancer. Therefore, our study focuses on the reliability, efficacy, and safety of lobaplatin as an intraperitoneal chemotherapeutic agent in stomach cancer.

After gastrectomy, one group of patients received lobaplatin IPC, and the other did not receive any IPC. There was no statistically significant difference in terms of loss of blood, duodenal stump/anastomotic leakage, intestinal obstruction, abdominal infection, or surgical site infection between the two groups. Two patients had peripheral blood leukocyte counts ~<3,500/mm^3^, but abnormal liver function tests or abnormal renal function tests were not present in any patient. Patients on HIPEC had an abnormal incidence that was comparable to non-HIPEC patients. Throughout the perioperative phase, no patients passed away. Both groups' postoperative hospital stays (7.7±1.5 and 7.5±1.3 days, p=0.423) were comparable [26]. It was additionally discovered that among HIPEC patients, the peritoneal cavity recurrence rate was significantly lower (4.9% versus 17.6%). The three-year disease-free survival (DFS) rate was higher in the HIPEC group (89.4%) than in the non-HIPEC group (73.9%), despite the fact that the estimated rate of three-year overall survival (OS) was not higher in the HIPEC group [27]. The results were similar to our study as none of our population developed abnormal RFTs and abnormal LFTs. In our population, there was only one case of peritoneal recurrence.

A group of 106 gastric cancer patients at T4 stage were treated with either radical gastric resection or prophylactic HIPEC. The postoperative platelet counts in the HIPEC group were significantly lower than those in the non-HIPEC group with no bleeding and similar rates of postoperative complications. Following surgery, postoperative (one month) levels of carcinoembryonic antigen (CEA), carbohydrate antigen (CA) 19-9 (CA19-9), and CA72-4 were significantly lower in the HIPEC group. At a median follow-up of 59.3 months, peritoneal recurrence was observed in three (5.5%) patients in the HIPEC group and 10 (18.2%) patients in the non-HIPEC group. Both groups showed comparable five-year overall survival (OS) rates (HIPEC group: 41.1%, non-HIPEC group: 34.5%). The HIPEC group had a significantly higher five-year disease-free survival rate than the non-HIPEC group [28]. The unmet need for additional efficacious treatment options to prevent peritoneal relapse after gastrectomy exists in patients with gastric cancer. Cytoreductive surgery, which includes peritoneal metastasis removal, can enhance overall survival and progression-free survival in some patients when paired with hyperthermic intraperitoneal chemotherapy. Patients with GC and peritoneal metastases who get intraperitoneal chemotherapy after having all original and metastatic tumors removed tend to have a better prognosis [29].

In our study, the age range of the population was 18-90 years. The mean age was 44.83 years with a median of 40.50 years. The mean BMI of our population was 33.31 kg/m^2^. Of the 30 patients, 18 were male, and the remaining 12 were female. Seventeen patients were at T3 stage, and 23 patients were at T4 stage. N3a was the most involved lymph node stage in our study population. The clinical stage of 14 patients was stage III, and 16 patients were at stage IV. The remaining postoperative complications in our study were also less, making lobaplatin a reliable, safe, and efficient agent for intraoperative intraperitoneal chemotherapy in gastric patients.

Limitations

Because of the small sample size of the study, the results cannot be generalized, and the data, being from a single center, might lack external validity. The results may not be applicable to the broader population. The retrospective nature of the study may introduce biases. The lack of a comparison group and limited time for follow-up are the other limitations. The potential confounding factors such as comorbid conditions, concomitant medication, and previous treatment could not be controlled due to the retrospective nature of the study. These confounding factors may influence the outcomes of the study.

## Conclusions

The study concludes that lobaplatin, when used in intraoperative chemotherapy for gastric cancer surgery, shows promising outcomes as a safe and effective treatment method that can lead to better patient results with few complications after surgery. However, the study's sample size is modest, emphasizing the need for more thorough research to confirm these findings over a wider demographic spectrum. Future research is required to fully define lobaplatin's therapeutic role and modify intraperitoneal chemotherapy methods for gastric cancer, with a particular emphasis on preventing peritoneal relapse and improving overall survival rates.

## References

[1] Brandl A, Prabhu A (2021). Intraperitoneal chemotherapy in the treatment of gastric cancer peritoneal metastases: an overview of common therapeutic regimens. J Gastrointest Oncol.

[2] Ajani JA, D'Amico TA, Bentrem DJ (2022). Gastric cancer, version 2.2022, NCCN Clinical Practice Guidelines in Oncology. J Natl Compr Canc Netw.

[3] (2020). The global, regional, and national burden of stomach cancer in 195 countries, 1990-2017: a systematic analysis for the Global Burden of Disease study 2017. Lancet Gastroenterol Hepatol.

[4] Yang L, Ying X, Liu S (2020). Gastric cancer: epidemiology, risk factors and prevention strategies. Chin J Cancer Res.

[5] Rawla P, Barsouk A (2019). Epidemiology of gastric cancer: global trends, risk factors and prevention. Prz Gastroenterol.

[6] Clinton SK, Giovannucci EL, Hursting SD (2020). The World Cancer Research Fund/American Institute for Cancer Research third expert report on diet, nutrition, physical activity, and cancer: impact and future directions. J Nutr.

[7] Chang CJ, Tu YK, Chen PC, Yang HY (2020). Talc exposure and risk of stomach cancer: systematic review and meta-analysis of occupational cohort studies. J Formos Med Assoc.

[8] Lin XJ, Wang CP, Liu XD (2014). Body mass index and risk of gastric cancer: a meta-analysis. Jpn J Clin Oncol.

[9] Mukkamalla SK, Recio-Boiles A, Babiker HM (2023). Gastric cancer. https://pubmed.ncbi.nlm.nih.gov/29083746/.

[10] Khan H, Johnston FM (2022). Current role for cytoreduction and HIPEC for gastric cancer with peritoneal disease. J Surg Oncol.

[11] Wagner AD, Syn NL, Moehler M (2017). Chemotherapy for advanced gastric cancer. Cochrane Database Syst Rev.

[12] Parray A, Gupta V, Chaudhari VA, Shrikhande SV, Bhandare MS (2021). Role of intraperitoneal chemotherapy in gastric cancer. Surg Prac Sci.

[13] Kim TH, Kim IH, Kang SJ (2023). Korean practice guidelines for gastric cancer 2022: an evidence-based, multidisciplinary approach. J Gastric Cancer.

[14] Yan K, Wu K, Yan L, Liang L, Yuan Y (2019). Efficacy of postoperative intraperitoneal hyperthermic perfusion chemotherapy with oxaliplatin + 5-fluorouracil in the treatment of gastric cancer patients with peritoneal carcinomatosis. J BUON.

[15] Denlinger CS, Sanft T, Moslehi JJ (2020). NCCN guidelines insights: survivorship, version 2.2020. J Natl Compr Canc Netw.

[16] Wang FH, Zhang XT, Li YF (2021). The Chinese Society of Clinical Oncology (CSCO): clinical guidelines for the diagnosis and treatment of gastric cancer, 2021. Cancer Commun (Lond).

[17] Muro K, Van Cutsem E, Narita Y (2019). Pan-Asian adapted ESMO Clinical Practice Guidelines for the management of patients with metastatic gastric cancer: a JSMO-ESMO initiative endorsed by CSCO, KSMO, MOS, SSO and TOS. Ann Oncol.

[18] Lei Z, Wang J, Li Z (2020). Hyperthermic intraperitoneal chemotherapy for gastric cancer with peritoneal metastasis: a multicenter propensity score-matched cohort study. Chin J Cancer Res.

[19] Chia DK, So JB (2020). Recent advances in intra-peritoneal chemotherapy for gastric cancer. J Gastric Cancer.

[20] Brenkman HJ, Päeva M, van Hillegersberg R, Ruurda JP, Haj Mohammad N (2019). Prophylactic hyperthermic intraperitoneal chemotherapy (HIPEC) for gastric cancer-a systematic review. J Clin Med.

[21] Desiderio J, Chao J, Melstrom L (2017). The 30-year experience-a meta-analysis of randomised and high-quality non-randomised studies of hyperthermic intraperitoneal chemotherapy in the treatment of gastric cancer. Eur J Cancer.

[22] Rau B, Lang H, Koenigsrainer A (2024). Effect of hyperthermic intraperitoneal chemotherapy on cytoreductive surgery in gastric cancer with synchronous peritoneal metastases: the phase III GASTRIPEC-I trial. J Clin Oncol.

[23] Wu HT, Peng KW, Ji ZH (2016). Cytoreductive surgery plus hyperthermic intraperitoneal chemotherapy with lobaplatin and docetaxel to treat synchronous peritoneal carcinomatosis from gastric cancer: results from a Chinese center. Eur J Surg Oncol.

[24] Ye J, Ren Y, Wei Z (2018). Nephrotoxicity and long-term survival investigations for patients with peritoneal carcinomatosis using hyperthermic intraperitoneal chemotherapy with cisplatin: a retrospective cohort study. Surg Oncol.

[25] Yin CY, Lin XL, Tian L, Ye M, Yang XY, Xiao XY (2014). Lobaplatin inhibits growth of gastric cancer cells by inducing apoptosis. World J Gastroenterol.

[26] Zhong Y, Zhang J, Bai X (2020). Lobaplatin in prophylactic hyperthermic intraperitoneal chemotherapy for advanced gastric cancer: safety and efficacy profiles. Cancer Manag Res.

[27] Zhong Y, Kang W, Hu H, Li W, Zhang J, Tian Y (2023). Lobaplatin-based prophylactic hyperthermic intraperitoneal chemotherapy for T4 gastric cancer patients: a retrospective clinical study. Front Oncol.

[28] Oya Y, Hayakawa Y, Koike K (2020). Tumor microenvironment in gastric cancers. Cancer Sci.

[29] Rau B, Feldbrügge L, Gronau F, Alberto Vilchez ME, Thuss-Patience P, Bonnot PE, Glehen O (2022). Indication of hyperthermic intraperitoneal chemotherapy in gastric cancer (Gastripec, Gastrichip). Visc Med.

